# Perinatal characteristics, older siblings, and risk of ankylosing spondylitis: a case–control study based on national registers

**DOI:** 10.1186/s13075-016-0917-1

**Published:** 2016-01-19

**Authors:** Ulf Lindström, Helena Forsblad-d’Elia, Johan Askling, Lars Erik Kristensen, Elisabeth Lie, Sofia Exarchou, Lennart Jacobsson

**Affiliations:** Department of Rheumatology and Inflammation Research, Institute of Medicine, Sahlgrenska Academy, University of Gothenburg, Guldhedsgatan 10A, 405 30 Gothenburg, Sweden; Institution of Public Health and Clinical Medicine/Rheumatology, Umeå University, Lasarettsgatan 7, 901 87 Umeå, Sweden; Rheumatology Unit and Clinical Epidemiology Unit, Department of Medicine Solna, Karolinska Institutet, Nobels väg 5, Solna, 171 76 Stockholm Sweden; The Parker Institute, Department of Rheumatology, Frederiksberg and Bispebjerg Hospital, Nordre Fasanvej 57, 2000 Frederiksberg, Denmark; Department of Rheumatology, Diakonhjemmet Hospital, Diakonveien 14, Vinderen, 0319 Oslo, Norway; Section of Rheumatology, Department of Clinical Sciences, Lund University, Barngatan 2B, 221 85 Lund, Sweden

**Keywords:** Ankylosing spondylitis, Epidemiology, Pathogenesis, Spondyloarthritis

## Abstract

**Background:**

The effect of circumstances and exposures early in life on the risk of developing ankylosing spondylitis (AS) is largely unknown. The purpose of this study was to determine whether perinatal characteristics predict development of AS.

**Methods:**

AS cases (n = 1960; 59 % men) were defined as listed with a diagnosis of AS at least once in the Swedish National Patient Register and registered in the Swedish Medical Birth Register (born ≥1973). Population controls were retrieved from the Swedish Population Register (n = 8378; mean 4.3 controls/case), matched on birth year, sex and county. Odds ratios (OR) for developing AS were determined through conditional logistic regression, with regard to: birth weight, birth order, season of birth, maternal age, gestational length, size for gestational age, type of birth, mode of delivery, congenital malformations, mothers’ country of birth, mothers’ civil status and size of delivery unit.

**Results:**

In the univariate analyses statistically significant increases in risk for developing AS were observed for having older siblings (OR 1.18; 95 % Cl 1.06–1.30). No association was observed for the remainder of analysed exposures, although there was a weak association with birth weight below 3000 g (OR 1.19; 95 % CI 1.04–1.37), though not for “low birth weight” <2500 g (OR 0.90; 95 % CI 0.70–1.16). The increase in risk associated with having older siblings was consistent in a multivariate analysis adjusting for possible confounders (OR 1.23; 95 % Cl 1.09–1.39). The direction and magnitude of the point estimates were also consistent in several sensitivity analyses and when stratifying by sex.

**Conclusions:**

Having older siblings was associated with an increased risk for developing AS. These results need to be repeated and confirmed in other cohorts.

**Electronic supplementary material:**

The online version of this article (doi:10.1186/s13075-016-0917-1) contains supplementary material, which is available to authorized users.

## Background

Ankylosing spondylitis (AS) is a chronic and progressive inflammatory disease with onset of symptoms before the age of 45 in the majority of cases, and an estimated 1.30–1.56 million prevalent cases in Europe [1, 2]. Other common inflammatory diseases, such as inflammatory bowel disease (IBD), psoriasis, and anterior uveitis are overrepresented in AS, suggesting shared aetiology [3]. The strong correlation between AS and the human leukocyte antigen (HLA) B27 has been extensively investigated [4–8]. Based on twin studies the majority of the susceptibility for AS can be attributed to genetic factors [3], but environmental factors are also likely to contribute to the risk [9, 10], a contribution that may differ depending on phenotype, sex, or age of onset. One proposed hypothesis states that infections may trigger the disease onset in genetically susceptible individuals [10], in similarity to the related disease reactive arthritis [11, 12]. Another hypothesis suggests that mechanical stress at the entheses may initiate inflammation and bone remodelling [13]. However, so far the evidence supporting the role of specific environmental risk factors in the development of AS has been limited.

Number of siblings is often applied as a proxy for exposure to childhood infections and has been shown to affect the risk for developing immune-related diseases [14]. Studies have found an inverse relationship between sibship size and risk for allergic diseases, such as childhood asthma and eczema [15–17]. This has been attributed to the “hygiene hypothesis”, postulating that an increased exposure to infections during childhood may lead to a lower risk for developing allergy [18]. Two previous studies, investigating the effect of birth order on the risk for developing AS have produced conflicting results [19, 20].

The relationship between exposures even earlier in life, such as factors influencing pregnancy and birth, and later development of disease has been established for diabetes and cardiovascular disease but it has also been implicated in several diseases characterised primarily by inflammation or autoimmunity [21]. Low birth weight has, for example, been associated with a decreased risk for later development of rheumatoid arthritis (RA), and high birth weight with an increased risk [22–24]. High birth weight may also be associated with an increased risk of Sjögren’s syndrome [25]. Similarly, season of birth has also been shown to be associated with variations in the risk for subsequent development of certain diseases. For example, children born during autumn and winter months have been shown to have a higher risk for food allergy (suggested to be related to UVB exposure and vitamin D) [26, 27]. Other studies have also indicated a variation in the risk for development of IBD, depending on season or month of birth [28, 29].

No large population-controlled studies have been performed investigating how circumstances and exposures early in life affect the risk of developing AS. The overarching aim of this study was to assess the role of environmental factors, specifically pregnancy and perinatal characteristics, in the pathogenesis of AS, with particular focus on birth order, birth weight and season of birth.

## Methods

### Setting and data sources

This is a case–control study, based on the Swedish national health care and population registers.

The Swedish National Patient Register (NPR) was initiated in 1964, as an inpatient register collecting administrative and medical data, including diagnoses registered according to the Swedish version of the International Classification of Diseases (ICD) [30] codes at discharge from hospital. Since 1987 the coverage is considered to be close to 100 % [31]. In 2001 an outpatient register was added for outpatient specialized care. The coverage is lower, approximately 80 %, mostly due to missing data from private care [32]. The NPR is considered to have a very high validity in general [33]. Primary care visits are not included in the NPR.

The Swedish population register, managed by Statistics Sweden, contains data on residence, migration and socioeconomic factors for all residents [34].

The Swedish Medical Birth Register (MBR) was initiated in 1973 and includes administrative and medical data related to pregnancy, partum and postpartum neonatal care [35]. It is compulsory for all health care providers involved in prenatal maternity care, delivery or neonatal care to report to the register.

The Prescribed Drug Register contains data on all drugs prescribed in Sweden since July 2005, e.g. date prescribed/dispensed, dose and Anatomical Therapeutic Chemical code (ATC code [36]). The Anti-Rheumatic Therapy in Sweden (ARTIS) Register is a national treatment register, aimed at monitoring disease activity and outcome and safety of pharmacological treatment (including biological drugs) in rheumatic diseases. All public and private rheumatology clinics report to the register, which has an estimated national coverage of 85–95 % for patients treated with tumour necrosis factor inhibitors (TNFi) [37, 38].

All the registers include the individuals personal identification number (PIN), given to all residents in Sweden, enabling cross-linking of different registers [39].

In this study the NPR was used to identify the AS cases and to retrieve data on AS-related inflammatory diseases 1973–2009. Matched controls (see below) were obtained from the population register and data on perinatal characteristics from the MBR. Data on pharmacological treatment were retrieved from the Prescribed Drug Register and the ARTIS register. All ICD and ATC codes used are presented in supplementary Table S1 (Additional file 1).

### Study population

AS cases were defined as all individuals registered in the MBR (born in Sweden ≥1973) who at least once up until 2009 had a visit listing an ICD code for AS (ICD-8: 712, 40, ICD-9: 720A, ICD-10: M45) registered in the NPR. We have previously performed a validation study of the AS diagnoses in the NPR and found good validity, with a positive predictive value of at least 80 % for fulfilling the modified New York criteria and 89–97 % fulfilling any of the commonly used classification criteria for spondyloarthritis (SpA) [40].

For each AS case five controls, matched on birth year, sex and county of residence (at the time of their first SpA diagnosis), were retrieved from the population register. Matching at the time of the first SpA diagnosis, rather than the first AS diagnosis, was chosen as the preferred method of matching since a number of cases were expected to first receive another SpA diagnosis before development and subsequent documentation of structural changes of the sacroiliac joints or spine, resulting in a diagnosis of AS. From the population register we also retrieved data on level of formal education and disposable income in 2008, which was used to compare socioeconomic status between cases and controls.

Data on AS-related disease manifestations, conventional synthetic disease-modifying anti-rheumatic drugs (csDMARDs) and TNFi were used for a stratification of disease severity for the AS cases in the sensitivity analyses: from the NPR we collected data on all registered visits to a non-primary care physician with an ICD code attributed to some of the AS-related inflammatory diseases (IBD, psoriasis, and anterior uveitis) for both cases and controls, from birth and up to 2 years after the first AS diagnosis for the index case (at the latest up until 31 December 2011). A 2-year lag period after the first diagnosis of AS was chosen to include patients diagnosed with IBD and psoriasis as a result of medical investigations initiated at the time of the first AS diagnosis.

For the AS cases data on pharmacological treatment, prescribed between 1 January 2011 and 31 December 2011, were obtained from the Prescribed Drug Register for the following csDMARDS: methotrexate and sulphasalazine; and for subcutaneously administered TNFi: etanercept, adalimumab, golimumab and certolizumab pegol. From the ARTIS we obtained data on registered treatment with infliximab for the same time period, since this intravenously administered TNFi is normally not prescribed through a pharmacy and thus not included in the Prescribed Drug Register.

### Exposure data

Variables associated with birth characteristics were retrieved from the MBR. The variables assessed were: maternal age, mother’s country of birth (Sweden, Nordic or other), mother’s civil status (mother married or living with father of the child versus other situation, as recorded in the MBR), number of older siblings, season of birth, mode of delivery (caesarean/vaginal), type of birth (single/multiple), birth weight, birth length, gestational length, birth weight for gestational age [41], congenital malformations, and maternal smoking during pregnancy. In order to adjust for systematic errors arising from possible variations in practice between delivery units, a variable coding for size of delivery unit (based on number of deliveries in the MBR during the study period) was also included. The categories used in the analyses are presented in Table 2. Birth weight was defined as low birth weight <2500 g, normal birth weight 2500–4200 g and high birth weight ≥4300 g. Additional definitions, using alternative cutoffs, were also analysed: <3310 g, 3310–3725 g and ≥3726 g (tertiles) as well as <3000 g, 3000–3999 g and ≥4000 g (to enable comparisons with previous similar studies [24]). Data on a recorded diagnosis of diabetes, among the mothers prior or during the pregnancy, were also obtained from the MBR.

### Statistics

Univariate odds ratios (ORs) were computed for the association between exposures (birth characteristics) and the outcome (a diagnosis of AS), by use of conditional logistic regression.

Birth weight and birth order were also assessed in a multivariate conditional logistic regression analysis, which also included possible confounders (gestational length, maternal age and type of birth (single/multiple)). Two measures of socioeconomic status (mother’s country of birth and civil status) as well as size of the delivery unit were also included. Season of birth was not entered in the multivariate analysis as the univariate analysis did not reveal any sign of association to development of AS.

The univariate and the main multivariate analyses were also stratified by sex and by birth year above or below the median.

Four sensitivity analyses, essentially subset analyses, were performed, using the same multivariate model as described above. First, excluding all cases and controls with a diagnosis of psoriasis, anterior uveitis, or IBD before or within 2 years after the first AS diagnosis of the index case (excluding 26 % of the entire study population). Second, restricting the analyses to only include cases treated with methotrexate, sulphasalazine, or TNFi during the last year of data collection (2011), limiting the analyses to a presumably more severe subgroup of AS (excluding 64 % of the entire study population). Third, limiting the analysis only to the cases who had received their AS diagnosis at least once from a specialist in rheumatology or internal medicine (excluding 27 % of the entire study population). Fourth, limiting the analysis only to cases who had received an AS diagnosis at least once in outpatient specialized care 2001–2009 (excluding 7 % of the entire study population). An additional analysis, based on only cases and controls still living in their county of birth at the time point of matching, was also performed to minimize the possible influence of migration between regions with different prevalences of ankylosing spondylitis.

Level of formal education and disposable income in 2008 were compared between cases and controls. Level of formal education was categorized as ≤9, 10–12 and >12 years of schooling and compared using a chi-square test. Disposable income in 2008 was only assessed for those ≥30 years old in 2008, an age when most individuals are assumed to have finalized their formal education, and in order to avoid extreme outliers we only included the 0.5^th^ to the 99.5^th^ percentile. Comparison of disposable income was performed using the *t* test. Comparisons of the proportion of mothers with diabetes, between cases and controls, were performed based on Fisher’s exact test.

SAS version 9.3 for Windows (SAS Institute, Cary, NC, USA) was used for aggregation of the data, and SPSS version 21 for Windows (IBM Corp., Armonk, NY, USA) was used for the statistical analyses.

### Ethical approval

The study was approved by the regional ethics committee in Stockholm, Sweden, 9 September 2011 (number: 2011/29-31/1) and conducted in compliance with the Declaration of Helsinki. Informed consent from individual patients/controls was not required.

## Results

### Cases and controls

We identified 1960 AS cases (59 % men) and 8377 matched population controls, included in the MBR (born in Sweden after 1972). A total of 95 % of the cases had ≥3 matched controls. Median age at inclusion (matched at first SpA diagnosis) was 25 years (25^th^percentile = 20; 75^th^ percentile = 28). Demographics, pharmacological treatment, AS-related inflammatory diseases and level of formal education and disposable income in 2008 are presented in Table 1. No statistically significant difference was observed between cases and controls with respect to level of formal education, disposable income or maternal diabetes prior or during pregnancy.

**Table 1:** Characteristics of the 1960 cases with ankylosing spondylitis and the 8377 matched population controls

Demographics	Cases n = 1960	Controls n = 8377
Women (%)	801 (41)	346 (41)
Year of birth median (Q1,Q3)	1979 (1975, 1984)	1979 (1975, 1984)
Age at first AS diagnosis median (Q1, Q3)	27 (22, 30)	NA
Living in same county at time of matching and at birth	1299 (66)	5571 (67)
AS-related inflammatory diseases^a^		
Anterior uveitis (%)	337 (17)	22 (0)
Inflammatory bowel disease (%)	108 (6)	58 (1)
Psoriasis (%)	89 (5)	49 (1)
Any of the manifestations above (%)	496 (25)	127 (2)
Rheumatic diagnoses before first AS diagnosis^b^		
Spondyloarthritis diagnosis^c^	627 (32)	NA
Any rheumatic diagnosis^d^	630 (32)	NA
Pharmacological treatment in 2011^e^		
TNF-alpha inhibitor (%)	494 (25)	NA
Methotrexate (%)	161 (8)	NA
Sulphasalazine (%)	217 (11)	NA
Any of the treatments above (%)	700 (36)	NA
Years of formal of education in 2008^f^		
0–9 years	309 (16)	1160 (14)
10–12 years	880 (45)	3705 (44)
>12 years	679 (35)	2993 (36)
Disposable income in 2008 £ mean (SD)^g^	16378 (6797)	16768 (7050)
Maternal diabetes (%)^h^	12 (0.6)	36 (0.4)

*AS* ankylosing spondylitis. *NA* no data available, *TNF* tumour necrosis factor

^a^AS-related inflammatory diseases are given as cumulative incidence until 2 years after first AS diagnosis for the cases and their matched controls

^b^Rheumatic diagnoses before first AS diagnosis are based on ICD codes in the National Patient Register

^c^Psoriatic arthritis, undifferentiated spondyloarthritis, reactive arthritis and spondyloarthritis associated with inflammatory bowel disease

^d^Any rheumatic disease, see Table S1 in Additional file 1

^e^Pharmacological treatment is based on data in the Prescribed Drug Register for prescriptions during 2011, or in the case of infliximab recoded in the Anti-Rheumatic Therapy in Sweden (ARTIS) register as used during 2011

^f^No statistically significant difference in level of formal education based on chi-square test (*p* = 0.115)

^g^Limited to those ≥30 years old in 2008 and the 0.5^th^ to the 99.5^th^ percentiles (cases = 953; controls = 3977), no statistically significant difference based on *t* test (*p* = 0.125)

^h^Maternal diabetes prior or during pregnancy, as recorded in the Medical Birth Register. No statistically significant difference based on Fisher’s exact test (*p* = 0.271)

### Univariate analyses

In the univariate analyses a statistically significant association with AS (Table 2) was observed for having older siblings (OR 1.18; 95 % confidence interval (CI) 1.06–1.30). There was no association between season of birth, or birth weight defined as <2500/2500–4200/≥4200 grams, and risk of AS. Categorizing birth weight according to previous similar studies [24] resulted in a weak but significant association for a birth weight <3000 g (OR 1.19; 95 % CI 1.04–1.37; 3000–4000 g: reference; >4000 g: OR 1.04; 95 % CI 0.91–1.19) and categorizing into tertiles resulted in similar, but non-significant point estimates (<3310 g: OR 1.11; 95 % CI 0.99–1.25; 3310–3725 g: reference; >3725 g: OR 1.03 95 % CI 0.91–1.65). For the remainder of the analysed variables there were no statistically significant associations with the outcome of developing AS, although the point estimate for multiple births (OR 1.35; 95 % CI 0.95–1.90) indicated an association, but based on very few events (men = 27 (2.3 %), women = 17 (2.1 %)). Further analysis of multiple births revealed that it was associated with development of AS only for men (OR 1.80; 95 % CI 1.14–2.84) and not for women (OR 0.95; 95 % CI 0.55–1.63). Due to lack of a unique identifier for the mothers, our data did not permit us to identify cases or controls belonging to the same multiple birth (e.g. twins), but eight possible twin pairs (born on the same day, at the same delivery unit) were identified, with no unexpected preponderance for either cases or controls. There was no association between maternal smoking and development of AS (OR 0.96; 95 % CI 0.78–1.17), although this was only available in cases born 1982 and later and in a total for 31 % of the cases. No interactions were observed between maternal smoking and birth weight or having older siblings.

**Table 2:** Birth characteristics for ankylosing spondylitis compared to matched population controls, with univariate odds ratios

	Cases (%)	Controls (%)	OR (95 % Cl)
	(n = 1960)	(n = 8377)	
Maternal age, years			
<25	643 (33)	2676 (32)	1.04 (0.92 to 1.17)
25–29	729 (37)	3156 (38)	1 (reference)
30–34	419 (21)	1815 (22)	1.00 (0.87 to 1.14)
35+	169 (9)	730 (9)	1.01 (0.84 to 1.21)
Missing	0	0	
Mothers’ civil status			
Mother married or living with father of the child	1322 (67)	5753 (67)	1 (reference)
Other	463 (24)	1896 (23)	1.06 (0.94 to 1.21)
Missing	175 (9)	728 (9)	
Mothers’ country of birth			
Sweden	1762 (90)	7550 (90)	1 (reference)
Nordic	107 (6)	385 (5)	1.17 (0.93 to 1.47)
Other	91 (5)	442 (5)	0.81 (0.64 to 1.03)
Missing	0	0	
Number of older siblings			
0	750 (38)	3550 (42)	1 (reference)
≥1	1210 (62)	4827 (58)	1.18 (1.06 to 1.30)
Missing	0	0	
Type of birth			
Singletons	1916 (98)	8236 (98)	1 (reference)
Multiple birth	44 (2)	141 (2)	1.35 (0.95 to 1.90)
Missing	0	0	
Mode of delivery			
Vaginal	1742 (89)	7544 (90)	1 (reference)
Caesarean	218 (11)	833 (10)	1.14 (0.97 to 1.33)
Missing	0	0	
Season of birth			
March-May	568 (29)	2354 (28)	1 (reference)
June-August	473 (24)	2073 (25)	0.95 (0.83 to 1.09)
September-November	416 (21)	1969 (24)	0.87 (0.76 to 1.00)
December-February	503 (26)	1981 (24)	1.04 (0.91 to 1.19)
Missing	0	0	
Birth weight, g			
<2500	76 (4)	355 (4)	0.90 (0.70 to 1.16)
2500–4200	1764 (90)	7476 (89)	1 (reference)
≥4200	113 (6)	536 (6)	0.90 (0.73 to 1.11)
Missing	7 (0)	10 (0)	
Gestational length, days			
≤258	111 (6)	455 (5)	1.02 (0.82 to 1.27)
259–293	1611 (82)	6846 (82)	1 (reference)
≥294	196 (10)	915 (11)	0.92 (0.78 to 1.08)
Missing	42 (2)	161 (2)	
Birth weight for gestational age^a^			
Small for gestational age	66 (3)	316 (4)	0.89 (0.68 to 1.17)
Appropriate for gestational age	1788 (91)	7652 (91)	1 (reference)
Large for gestational age	49 (3)	227 (3)	0.95 (0.69 to 1.31)
Missing	57 (3)	183 (2)	
Congenital malformation			
No	1829 (93)	7760 (93)	1 (reference)
Yes	75 (4)	354 (4)	0.90 (0.70 to 1.17)
Missing	57 (3)	182 (2)	
Size of delivery unit (no of births ≥1973)			
≥300	484 (25)	2087 (25)	0.91 (0.76 to 1.09)
200–299	581 (30)	2502 (30)	0.92 (0.78 to 1.09)
100–199	598 (31)	2471 (30)	1 (reference)
<100	297 (15)	1317 (16)	0.93 (0.77 to 1.13)
Maternal smoking^b^			
Not smoking during pregnancy	170 (9)	769 (9)	1 (reference)
Smoking during pregnancy	440 (22)	1896 (23)	0.96 (0.78 to 1.17)
Missing	1350 (69)	5713 (68)	

Odds ratios (ORs) determined through conditional logistic regression analyses

^a^Multiple births excluded since reference intervals are not applicable

^b^Only available from 1982, when recording of maternal smoking was initiated

### Multivariate analyses

**Fig. 1:**
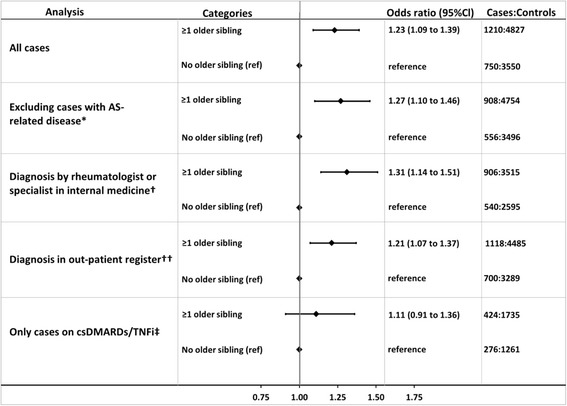
Odds ratios for developing ankylosing spondylitis with regard to having older siblings. Forest plot presenting odds ratios with 95 % confidence intervals for being diagnosed with ankylosing spondylitis, with regard to having older siblings, in a multivariate conditional logistic regression analysis and four sensitivity analyses. All the analyses were adjusted for birth weight, having older siblings, mother’s birth country, mother’s civil status, maternal age, gestational length, type of birth (single/multiple) and size of delivery unit. ^*^All cases with an ICD code for psoriasis, anterior uveitis or inflammatory bowel disease before or within 2 years of their AS diagnosis, and their respective controls are excluded. ^†^Including only cases, with their respective controls, who had received ≥1 AS diagnosis at a clinic of rheumatology or internal medicine. ^††^Including only cases with their respective controls who had received ≥1 AS diagnosis in the outpatient register 2001–2011. ^‡^Including only cases, with respective controls who were treated with csDMARDs (methotrexate and sulphasalazine) and/or TNFi during any part of 2011. *AS* ankylosing spondylitis, *csDMARDs* conventional synthetic disease-modifying anti-rheumatic drugs, *ICD* International Classification of Diseases, *TNFi* tumour necrosis factor inhibitor

The main multivariate analysis (Fig. 1) confirmed the associations observed in the univariate analyses. The ORs for being diagnosed with AS compared to controls were 1.23 (95 % CI 1.09–1.39) for having older siblings versus not. The ORs for birth weight were also similar in the multivariate analysis, compared to the univariate. None of the other exposures in the multivariate analysis were significantly associated with the development of AS and no point estimates except for multiple births (OR 1.25; 95 % CI 0.84–1.84) indicated a possible association.

### Sensitivity analyses

Overall, the OR for having older siblings was similar in all sensitivity analyses (Fig. 1), to those in the main analyses. Stratifying the univariate and multivariate analyses by sex and median birth year resulted in overall similar point estimates as in the stratified univariate analyses, supplementary Table S2 (Additional file 2). Multivariate analysis, only including cases and controls still living in their birth county, at the time of matching, revealed no important differences to the main analysis.

## Discussion

### Principal findings

In this study having older siblings was associated with an increased risk of receiving a diagnosis of AS. There was also a weak increase in risk associated with a birth weight <3000 g, which could not be confirmed in our primary stratifications of birth weight. We found no association between, season of birth, type of birth, mode of delivery, congenital malformations, gestational length or maternal age, and later being diagnosed with AS.

### Comparison to previous studies

Birth weight has to our knowledge not been studied as a risk factor for being diagnosed with AS. For IBD, which is associated with AS, one case–control study indicated that cases developing IBD had significantly lower birth weight compared to controls [42], which in that study was probably related to a significantly higher frequency of preterm birth. In our study there was no association between neither birth weight nor gestational length and development of AS in the univariate or any of the multivariate analyses, using the definition for low birth weight set by the World Health Association [43]. However, when using the alternative stratification of birth weight (<3000 g, 3000–4000 g, >4000 g), there was a slight increase in risk for birth weight <3000 g, and similarly so when birth weight was categorized into tertiles. This suggests differences in the distribution of birth weight between those later developing AS compared to controls, although the biological importance of this difference is unclear. Birth weight, in itself, is influenced by a multitude of factors, including gestational length, maternal nutrition [44], maternal diabetes [45], exposure to toxins such as maternal smoking [46], maternal age, sex of the child, socioeconomic status, and birth order [47, 48]. Adjusting for maternal age, gestational length, birth order, civil status of the mothers and type of birth, in the multivariate analyses did not affect the point estimates for birth weight. Maternal diabetes was not linked to the risk of AS in the offspring.

Low birth order was, in a previous study comparing 162 AS cases with their healthy siblings, a risk factor for developing AS [19]. In contrast to this a much larger register-based study of AS cases (4517 patients in the Bath AS register), investigating the position of birth within the family found no statistically significant effect of birth order on the risk of developing AS [20]. Due to their methodology, both of these studies only examined families with more than one child, in order to assess the effect of birth order. In our national case–control study, also including cases without siblings, having older siblings was significantly associated with an increased risk of later receiving a diagnosis of AS. Studies of IBD have indicated that having older siblings may be associated with an increased risk for ulcerative colitis, while having younger siblings may be associated with a decrease in risk for Crohn’s disease [49], although results are conflicting [50]. In previous studies, it has been shown that having older siblings constitute a risk for exposure to infections at a very young age [16], and it has been argued that having younger sibling may constitute a risk for exposure to infections during the later years of childhood [14]. In our study the available data did not allow us to assess the effect of having younger siblings. For the related disease – reactive arthritis – the direct pathogenic and temporal association with infectious diseases is well known [11, 12], and for IBD exposure to enteric infections is known to affect the risk of developing both Crohn’s disease and ulcerative colitis [50, 51]. Considering the conflicting results concerning the effect of birth order on the risk for developing AS in the current and the two previous studies [19, 20], further studies are needed, preferably also including information regarding type, frequency and severity of infections.

### Limitations and strengths of this study

Some limitations should be considered. First, misclassification of cases may be a problem when using health care registers for identification. Previous validation studies performed by our group have, however, demonstrated high validity for ICD codes for AS in the Swedish registers [40]. Furthermore, the proportion of men and women and the frequency of treatment with TNFi were very similar to that found in previous clinical studies on AS in Sweden [52], and the frequencies of IBD, psoriasis and anterior uveitis were also similar to other AS cohorts [53] (although the gender distribution was more equal than in some other cohorts [1, 53], which may to some extent be due to the inclusion of cases with non-radiographic axial SpA in the AS group, as indicated in the validation study [40]), supporting the validity of the case identification. Second, residual or unmeasured confounding could be an issue in all observational studies. We were able to adjust for socioeconomic status to some extent in our main multivariate analysis, and there was no statistically significant difference in level of formal education and disposable income in 2008 between cases and controls. However, other important factors, such as the family income at birth, mother’s body mass index (BMI) and the parent’s health status (e.g. ankylosing spondylitis), could not be taken into account in the analyses. Third, maternal smoking was only available for 32 % of the cases and during a limited time period. In itself it did not affect the risk of developing AS in the univariate analyses, but due to the limitations in the available data it could not be included in the multivariate analysis. Since smoking is known to be related to both low birth weight [46] and to predict radiographic progression in AS [54] the effect of maternal smoking, or exposure to smoking during childhood, on the risk for developing AS needs to be studied further. Fourth, due to the limited time frame of the study, patients with a long diagnostic delay would not be identified and included as cases [40, 55]. Fifth, using birth order as a possible proxy for childhood infections is inferior to directly recording actual infectious events, which was not possible for the cases/controls in this study.

Our study also has several strengths. First, this is the only study of this research question in AS comparing cases to population controls. Second, it is based on national health care registers, capturing the vast majority of AS cases in Sweden [56], which resulted in relatively large numbers and few missing data for most variables and therefore greater generalizability. This also decreases the risk of selection bias, which may have influenced the results in the previous studies of the effect of birth order on the risk for AS [19, 20]. Third, the consistent magnitudes and directions of the point estimates in the sensitivity analyses suggest that our results were reasonably robust.

## Conclusions

Our results point to environmental factors as possible predictors for development of AS. More specifically, we found that having older siblings increased the risk for a later diagnosis of AS, indicating that factors related to childhood exposure may be of importance in the disease pathogenesis.

## Additional files

Additional file 1:
**ICD and ATC codes used in the study.** (DOCX 16 kb) Additional file 2:
**Regression analyses stratified by sex and birth year.** (DOCX 20 kb)

## Abbreviations

ARTIS: Anti-rheumatic Therapy in Sweden
AS: Ankylosing spondylitis
ATC: Anatomical Therapeutic Chemical
CI: Confidence interval
csDMARDs: Conventional synthetic disease-modifying anti-rheumatic drugs
HLA: Human leukocyte antigen
IBD: Inflammatory bowel disease
ICD: International Classification of Diseases
MBR: Medical Birth Register
NPR: National Patient Register
OR: Odds ratio
PIN: Personal identification number
RA: Rheumatoid arthritis
SpA: Spondyloarthritis
TNFi: Tumour necrosis factor alpha inhibitor
