# Supplementary material for: Perinatal characteristics, older siblings, and risk of ankylosing spondylitis: a case–control study based on national registers
Source: Arthritis Res Ther. 2016 Jan 19;18:16. doi: 10.1186/s13075-016-0917-1 (PMC4718040; doi:10.1186/s13075-016-0917-1)
Supplement: Additional file 1: — ICD and ATC codes used in the study. (DOCX 16 kb) [file 13075_2016_917_MOESM1_ESM.docx]

Supplementary table S1.

Title:The different codes used in the study.

Legend: The codes for diseases are according to the different versions of the International Classification of Diseases (ICD) and the codes for drugs according to the Anatomical Therapeutic Chemical (ATC) system.

| Spondyloarthritis: | ICD-8 | ICD-9 | ICD-10 |
| --- | --- | --- | --- |
| Ankylosing spondylitis | 712,40 | 720A | M45 |
| Psoriatic arthritis | 696,00 | 696A, 713D | L405, M070, M071, M072, M073 |
| SpA associated with IBD |  |  | M074-M075 |
| Reactive Arthritis |  | 711B, 711D-711W | M013-M029, M03 |
| Undifferentiated SpA | 713,13; 726,99 | 720B, 720C, 720W, 720X | M460, M461, M468, M469 |
| Other rheumatic diseases: | | | |
| Systemic Lupus Erythematosus | 734,10 | 710A | M320, M321, M328, M329 |
| Juvenile Arthritis | 712,0 | 714D | M08, M09 |
| Rheumatoid arthritis | 712,10; 712,20; 712,38; 712,39 | 714A, 714B, 714C, 714W, 719D | M05, M060, M062, M063, M068, M069, M123 |
| Polyarthritis | 715,99 | 714X, 716F | M130 |
| Diseases related to ankylosing spondylitis: | | | |
| IBD | 563.00, 563.10, 569.02 | 555-556 | K50-K51 |
| Psoriasis | 696 | 696 | L40 |
| Anterior uveitis (iridocyclitis) |  | 364A-364B | H20, H221 |
| Pharmacological treatment: | ATC-code | | |
| Sulphasalazine | A07EC01 | conventional synthetic disease modifying anti-rheumatic drugs (csDMARDs) | |
| Methotrexate | L01BA01, L04AX03 |  |  |
| Etanercept | L04AB01 |  |  |
| Infliximab | L04AB02 |  |  |
| Adalimumab | L04AB04 | TNF- inhibitors (TNFi) |  |
| Certolizumabpegol | L04AB05 |  |  |
| Golimumab | L04AB06 |  |  |
| SpA = spondyloarthritis, IBD = inflammatory bowel disease. | | | |
|  | | | |
