# Supplementary material for: Perinatal characteristics, older siblings, and risk of ankylosing spondylitis: a case–control study based on national registers
Source: Arthritis Res Ther. 2016 Jan 19;18:16. doi: 10.1186/s13075-016-0917-1 (PMC4718040; doi:10.1186/s13075-016-0917-1)
Supplement: Additional file 2: — Regression analyses stratified by sex and birth year. (DOCX 20 kb) [file 13075_2016_917_MOESM2_ESM.docx]

Supplementary table S2

Title: Univariate and multivariate analyses stratified by sex and median birth year.

Legend: Presented results are limited to birth weight, season of birth and sibship size exposures. All multivariate analyses were in addition also adjusted for mothers’ birth country, mothers’ civil status, maternal age, gestational length, type of birth and size of delivery unit. Missing data not presented (see table 2).

|  | Men (cases n = 1159) | | | Women (cases n = 801) | | |
| --- | --- | --- | --- | --- | --- | --- |
| Univariate | Cases n(%) | Controls n(%) | Odds ratio (95% CI) | Cases n(%) | Controls n(%) | Odds ratio (95%CI) |
| No older siblings | 440 (38) | 2104 (42) | 1 (reference) | 310 (39) | 1446 (42) | 1 (reference) |
| ≥1 older sibling | 719 (62) | 2857 (58) | 1.19 (1.04 to 1.36) | 491 (61) | 1970 (58) | 1.16 (0.99 to 1.36) |
| Birth weight <2500 | 41 (4) | 196 (4) | 1.21 (1.00 to 1.45) | 35 (4) | 159 (5) | 1.17 (0.96 to 1.42) |
| Birth weight 2500-4200 | 1025 (88) | 4378 (88) | 1 (reference) | 739 (93) | 3099 (91) | 1 (reference) |
| Birth weight ≥4300 | 89 (8) | 382 (8) | 1.13 (0.96 to 1.33) | 24 (3) | 154 (5) | 0.86 (0.68 to 1.11) |
| March-May | 322 (28) | 1402 (28) | 1.00 (0.84 to 1.20) | 246 (31) | 952 (28) | 1.12 (0.91 to 1.39) |
| June-August | 285 (25) | 1245 (25) | 1 (reference) | 188 (24) | 828 (24) | 1 (reference) |
| September-November | 240 (21) | 1124 (23) | 0.92 (0.76 to 1.11) | 176 (22) | 845 (25) | 0.91 (0.73 to 1.15) |
| December-February | 312 (27) | 1190 (24) | 1.12 (0.94 to 1.35) | 191 (24) | 791 (23) | 1.05 (0.84 to 1.32) |
| Multivariate |  |  |  |  |  |  |
| No older siblings | 426 (38) | 2060 (42) | 1 (reference) | 299 (38) | 1406 (42) | 1 (reference) |
| ≥1 older sibling | 705 (62) | 2811 (58) | 1.25 (1.07 to 1.46) | 481 (62) | 1930 (58) | 1.20 (0.99 to 1.45) |
| Birth weight <2500 | 40 (4) | 195 (4) | 0.69 (0.0.44 to 1.08) | 35 (5) | 156 (5) | 0.88 (0.54 to 1.42) |
| Birth weight 2500-4200 | 1003 (89) | 4308 (88) | 1 (reference) | 723 (93) | 3035 (91) | 1 (reference) |
| Birth weight ≥4300 | 88 (8) | 368 (8) | 1.07 (0.83 to 1.39) | 22 (3) | 146 (4) | 0.66 (0.40 to 1.09) |
| Univariate | Birth year ≤ median (1979) (cases n=1067) | | | Birth year > median (1979) (cases n=893) | | |
| No older siblings | 390 (37) | 1935 (43) | 1 (reference) | 360 (40) | 1615 (42) | 1 (reference) |
| ≥1 older sibling | 677 (63) | 2576 (57) | 1.30 (1.13 to 1.49) | 533 (60) | 2251 (58) | 1.05 (0.90 to 1.22) |
| Birth weight <2500 | 37 (4) | 192 (4) | 0.80 (0.56 to 1.12) | 39 (4) | 163 (4) | 1.02 (0.71 to 1.34) |
| Birth weight 2500-4200 | 972 (91) | 4028 (89) | 1 (reference) | 792 (89)) | 3449 (89) | 1 (reference) |
| Birth weight ≥4300 | 58 (5) | 292 (7) | 0.83 (0.62 to 1.12) | 55 (6) | 244 (6) | 0.99 (0.72 to 1.34) |
| March-May | 297 (28) | 1290 (29) | 0.96 (0.80 to 1.16) | 271 (30) | 1064 (28) | 1.17 (0.96 to 1.44) |
| June-August | 265 (25) | 1111 (25) | 1 (reference) | 208 (23) | 962 (25) | 1 (reference) |
| September-November | 227 (21) | 1039 (23) | 0.91 (0.75 to 1.11) | 189 (21) | 930 (24) | 0.92 (0.74 to 1.15) |
| December-February | 278 (26) | 1071 (24) | 1.07 (0.89 to 1.30) | 225 (25) | 910 (24) | 1.12 (0.91 to 1.39) |
| Multivariate |  |  |  |  |  |  |
| No older siblings | 378 (36) | 1891 (43) | 1 (reference) | 347 (40) | 1575 (42) | 1 (reference) |
| ≥1 older sibling | 661 (64) | 2531 (57) | 1.40 (1.19 to 1.65) | 525 (60) | 2211 (58) | 1.04 (0.86 to 1.24) |
| Birth weight <2500 | 37 (4) | 189 (4) | 0.76 (0.50 to 1.18) | 38 (4) | 162 (4) | 0.80 (0.49 to 1.32) |
| Birth weight 2500-4200 | 946 (91) | 3952 (89) | 1 (reference) | 780 (89) | 3391 (90) | 1 (reference) |
| Birth weight ≥4300 | 56 (5) | 281 (6) | 0.82 (0.60 to 1.13) | 54 (6) | 233 (6) | 1.19 (0.85 to 1.67) |
